# Pre-immunotherapy radiotherapy enhanced the efficacy of multi-line sintilimab in unresectable advanced esophageal squamous cell carcinoma

**DOI:** 10.3389/fimmu.2023.960339

**Published:** 2023-02-20

**Authors:** Shuhui Xu, Xianxing Xu, Hui Zhu

**Affiliations:** ^1^ Department of Radiation Oncology, Shandong Cancer Hospital and Institute, Shandong First Medical University and Shandong Academy of Medical Sciences, Jinan, Shandong, China; ^2^ Department of Joint Surgery, Shandong Provincial Hospital Affiliated to Shandong First Medical University, Jinan, Shandong, China

**Keywords:** pre-immunotherapy, immunotherapy, sintilimab, esophageal squamous cell carcinoma, efficacy

## Abstract

**Background:**

The use of immunotherapy for the treatment of esophageal squamous cell carcinoma (ESCC) is gradually increasing. In this retrospective study, we evaluated the efficacy and explored potential factors of prognosis in multi-line sintilimab for unresectable advanced ESCC.

**Methods:**

All pathological specimens were available from our Department of Pathology. We performed PD-L1 immunohistochemical staining of surgical or puncture specimens from 133 patients. We evaluated the efficacy of multi-line sintilimab and found potential factors according to multivariate analysis. We assessed the relationship between radiotherapy and immunotherapy, and according to whether patients had received radiotherapy within 3 months prior to immunotherapy, we attempted to analyze differences in progression-free survival (PFS) and overall survival (OS).

**Results:**

A total of 133 patients were enrolled in this retrospective study between January 2019 and December 2021. The median follow-up was 16.1 months. All patients were treated with at least two cycles of sintilimab. Of all patients, a total of 74 experienced disease progression, with a median progression-free survival of 9.0 months (95% CI 7.701–10.299). We found that pre-immunotherapy radiotherapy was a possible predictor that affected the prognosis of multi-line sintilimab and that 3 months was a significant cutoff. A total of 128 patients (96.2%) had received radiotherapy prior to immunotherapy. Of those patients, 89 (66.9%) had received radiation therapy within 3 months prior to immunotherapy. PFS was considerably longer in patients who were treated within 3 months of radiotherapy than in patients who did not receive radiation therapy within 3 months of radiation therapy prior to immunotherapy (median progression-free survival 10.0 months [95% CI 8.030–11.970] *vs.* 5.0 months [95% CI 2.755–7.245]). Among all patients, the median overall survival was 14.9 months (95% CI 12.558–17.242). Overall survival was significantly longer in patients who had previously received radiotherapy within 3 months prior to immunotherapy than in those who had not (median overall survival 15.3 months [95% CI 13.724–16.876] *vs.* 12.2 months [10.001–14.399].

**Conclusion:**

Based on this retrospective study, sintilimab is a significant option for patients with unresectable advanced ESCC who have been previously treated, and pre-immunotherapy radiotherapy within 3 months enhanced the efficacy.

## 1 Introduction

Esophageal cancer (EC) is the primary cancer-related cause of death worldwide (1). Esophageal squamous cell carcinoma (ESCC) is one of the main typical epidemiological subtypes of EC, 95% of EC cases in China are ESCC, and 60% to 70% of patients have already reached a locally advanced or late stage at their first consultation (2, 3). For patients in an inoperable stage, it is hoped that the combination of chemotherapy and radiotherapy will improve the positive outcome of either treatment alone. These schemes aim to stabilize the patient’s disease progression and improve the prognosis but, unfortunately, do not cure or control the disease progression in the long term. Therefore, there is an unmet need for the development of more treatment options to treat refractory or relapsed advanced ESCC.

In recent years, significant efforts have been made to improve the prognosis of advanced ESCC patients. Among the studies of immune checkpoint inhibitors (ICIs), most focus on anti-programmed cell death protein 1(PD-1)/programmed cell-death protein 1 ligand 1 (PD-L1) antibodies (4–7). In the ATTRACTION-3 study, nivolumab demonstrated significant efficacy in unresectable advanced or recurrent ESCC (6). The KEYNOTE-028 and KEYNOTE-180 studies also proved the efficacy and safety of pembrolizumab in the treatment of advanced EC (8, 9). The results of the clinical trial showed that anti-PD-1 antibodies demonstrated promising anti-tumor effects in patients with advanced ESCC (10). These studies have revolutionized the therapeutic strategy for advanced ESCC. Sintilimab is a humanized IgG4 monoclonal antibody that binds to PD-1, targeting the interaction between PD-1 receptors and their ligands to block them, and effectively improves the function of T cells, enhancing immune surveillance and eliminating the ability of T cells in the tumor to produce the tumor immune response. Although the evidence of sintilimab in first-line treatment is strong in resectable advanced ESCC (11, 12), there have been limited reports of unresectable advanced ESCC patients who received multi-line sintilimab to date.

The combination of radiotherapy and PD-L1 blockade has shown promising responses in many tumors with multi-line treatments. Previous studies have shown that radiotherapy induces an organismal immune response that allows patients to escape from immunosuppression after receiving radiotherapy, which can promote systemic anti-tumor immune activation and upregulation of PD-L1 expression on tumor cells (13–16). Radiotherapy is also a double-edged sword, capable of producing both immune stimulation and immunosuppression. There is growing evidence that radiotherapy enhances the intrinsic and adaptive immune responses against tumors, thereby reducing immunosuppression. Therefore, in this retrospective study, we evaluated the efficacy of sintilimab in unresectable advanced esophageal squamous cell carcinoma and investigated whether radiotherapy within 3 months before immunotherapy could improve the effectiveness of multi-line sintilimab in unresectable advanced ESCC.

## 2 Materials and methods

### 2.1 Data collection

We retrospectively included the medical records of 133 unresectable advanced ESCC patients from Shandong Cancer Hospital between January 2019 and December 2021. The inclusion criteria were as follows: 1) confirmed unresectable advanced ESCC, 2) complete imaging reports before and after immunotherapy, 3) at least one measurable lesion, 4) available histological samples were taken in our hospital, 5) patients received sintilimab as third-line or multi-line treatment, 6) patients did not receive any immunotherapy treatment before sintilimab, 7) all patients were treated with at least two cycles of sintilimab, and 8) the Eastern Cooperative Oncology Group (ECOG) score was ≥1. Data regarding clinical features, disease characteristics, previous treatments, disease response, and outcomes were collected. All patients underwent a complete physical examination and complete history collection. All pathological diagnoses were confirmed by pathologists in our department. TNM stage was determined according to the eighth American Joint Committee on Cancer guidelines. The study was conducted in accordance with the Declaration of Helsinki and International Good Clinical Practice Guidelines (17).

### 2.2 PD-L1 expression and radiotherapy

**Figure 1:**
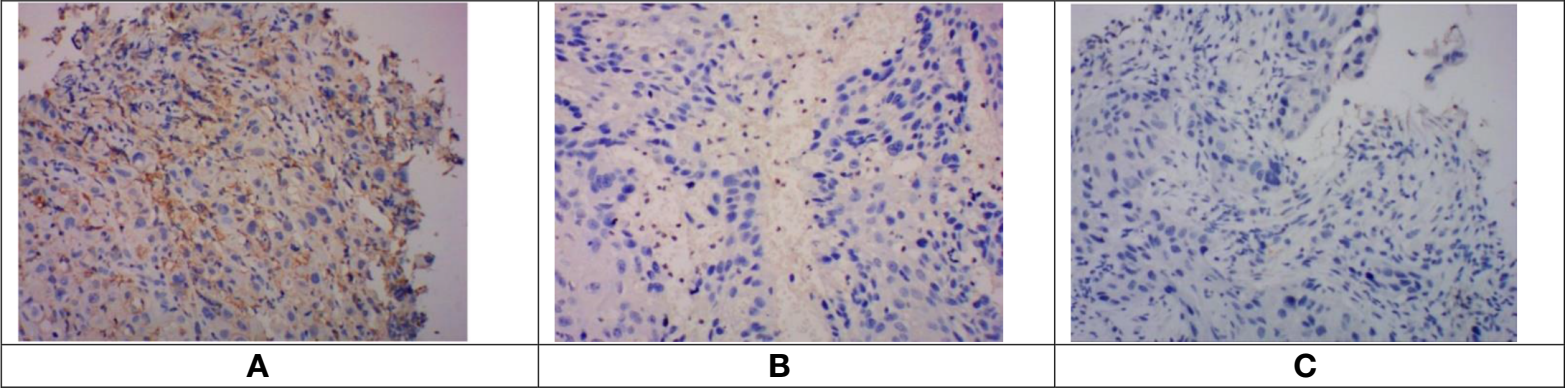
Immunohistochemical staining of PD-L1 in esophageal squamous cell carcinoma tissues. Images were obtained from three different patients. **(A)**. CPS > 10. **(B)** 1 < CPS 5 < 10. **(C)** CPS < 1 (negative of PD-L1 expression). PD-L1, programmed cell death protein ligand 1; CPS, combined positive score.

Tumor tissue samples from 133 participants were obtained for PD-L1 expression by immunohistochemistry. In this study, the measure of PD-L1 expression analysis was the combined positive score (CPS), defined as the sum of the number of PD-L1 stained cells, including tumor cells, lymphocytes, and macrophages, divided by the total number of tumor cells multiplied by 100. Expression of at least 1% was considered positive. PD-L1 was expressed both on the cell membrane and in the cytoplasm. In this retrospective study, PD-L1 expression showed heterogeneity in 133 patients (**Figure 1**).

The number of patients treated with radiotherapy within 1 month before immunotherapy was small and had no statistical significance. After a retrospective analysis of all patients, we found that 3 months could be used as the cutoff value of the interval between radiotherapy and immunotherapy. Based on that and to reduce the impact of other treatments on immunotherapy, patients were recorded as having received radiotherapy if they had received radiotherapy within 3 months prior to their first dose of sintilimab. We considered radiation therapy (RT) and sintilimab interval times beyond 3 months or no radiation as the same status, and these patients were classified in the group that did not receive radiotherapy. Based on the extensive data that were collected, local radiotherapy stimulated a systemic immune response.

### 2.3 Clinical response assessment

All patients were regularly followed up until the deadline (December 2021) or death by using clinical records. Follow-up visits were scheduled every 3 months. Progressive disease was verified by imaging review. Based on the Response Evaluation Criteria in Solid Tumors version 1.1, our study assessed objective tumor response by computed tomography (CT) scan (18). The CT before immunotherapy was used as a baseline, after which efficacy was assessed every 8 weeks, with the categories of responses being: complete response (CR), partial response (PR), stable disease (SD), or disease progression (PD). All patients were monitored regularly for possible adverse events (AEs). AEs were evaluated during the entire course of immunotherapy and up to 30 days after immunotherapy and were graded 0–4 in accordance with the National Cancer Institute Common Terminology Criteria for Adverse Events (version 4.0). The primary endpoints of this study were progression-free survival (PFS) and overall survival (OS). PFS was calculated from the date of initial treatment with sintilimab to the date of progression or death. OS was defined as the time from the first dose of sintilimab to the date of death from any cause. The secondary endpoints were objective response rate (ORR) (the percentage of patients with a confirmed complete or partial response) and disease control rate (DCR) (the proportion of patients with CR, PR, or SD in all cohorts receiving sintilimab).

### 2.4 Statistical analysis

Patients who lacked any of the indicators we needed were excluded from the study. We performed a multifactorial analysis with a Cox proportional hazards model, which was used to find possible predictors that affect prognosis. We used the χ^2^ test and Fisher’s exact test to compare the differences in baseline characteristics of patients between subgroups. We estimated progression-free survival, overall survival, median survival, and 95% CIs using the Kaplan–Meier analysis and compared subgroups (those who had received radiotherapy and those who had not received radiotherapy within 3 months before immunization) using the log-rank test. We used log-rank tests to detect the significant differences. The results with a p-value <0.05 were considered statistically significant.

## 3 Results

### 3.1 Patient characteristics

Between January 2019 and December 2021, 133 patients were assessed in this retrospective study. The clinical characteristics of both cohorts are presented in **Table 1**. Of the 133 patients tested, the median patient age was 65 years (range 48–78). Out of these, 24 patients (15.7%) had stage III disease, and 129 (84.3%) had stage IV disease. A total of 133 patients had samples that were evaluated for PD-L1 expression, and the median CPS was 15. A total of 33 (24.8%) had a CPS of 1% or greater, including 24 (18.0%) who had a CPS of 10% or greater. All patients received previous therapy before immunotherapy. A total of 133 patients were treated with systematic chemotherapy schemes before immunotherapy. Only five patients did not receive radiotherapy before immunotherapy. A total of 89 patients were treated with radiotherapy within 3 months prior to immunotherapy.

**Table 1:** Patient characteristics at baseline (N = 133).

Characteristics	N%	%
Sex		
Male	100	75.2
Female	33	24.8
Age (years) (median 65)		
<65	73	54.9
≥65	60	45.1
ECOG performance status score		
0	69	51.9
1	64	48.1
Tumor location		
Upper thoracic	41	30.8
Middle thoracic	55	41.4
Lower thoracic	37	27.8
T stage		
≤3	97	72.9
4	36	27.1
N stage		
≤2	78	58.6
3	55	41.4
M stage		
0	65	48.9
1	68	51.1
PD-L1 status		
Negative	100	75.2
Positive	33	24.8
PD-L1 expression (CPS)		
<1	100	75.2
1–10	9	6.8
>10	24	18.0
Previous chemotherapy schemes before immunotherapy	133	100
Only chemotherapy	5	3.8
Before radiotherapy	82	61.7
Concurrent radiotherapy	55	41.4
Sequential chemotherapy	44	33.1
Previous radiotherapy before immunotherapy	128	96.2
No radiotherapy	5	3.8
In 3 months	89	66.9
Beyond 3 months	39	29.3
Immunotherapy scheme		
Sintilimab alone	34	25.6
Sintilimab and chemotherapy	99	74.4
Immunotherapy-related toxicities		
White blood cell count decreased	49	36.8
Anemia	26	19.5
Rash	12	9.0
Aminotransferase increased	7	5.3
Diarrhea	4	3.0
Asthenia	1	0.8
Death	66	49.6

ECOG, Eastern Cooperative Oncology Group; CPS, combined positive score; PD-L1, programmed cell death protein ligand 1.

### 3.2 Potential factors in efficacy enhancement

**Figure 2:**
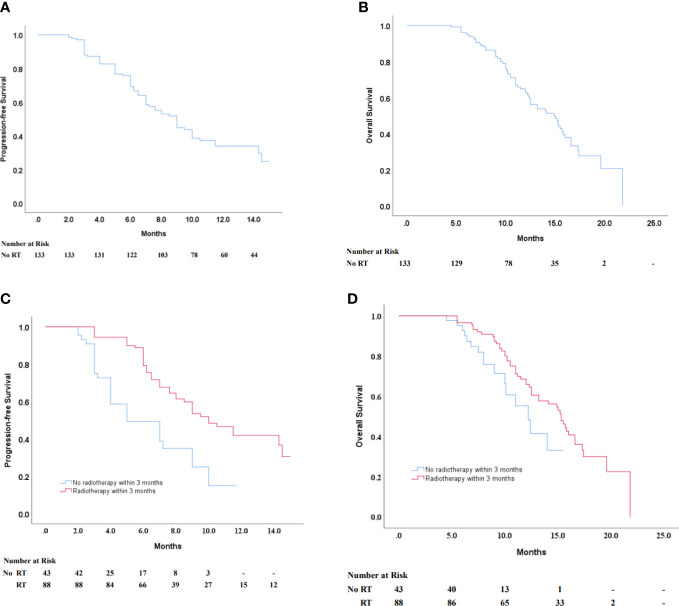
**(A, B)** Kaplan–Maier survival curves of PFS and OS of ESCC patients in all patients. Progression-free survival according to their history of radiotherapy within 3 months before immunotherapy **(C)** in all patients regardless of PD-L1 expression status **(D)** in a negative PD-L1 expression status. PFS, progression-free survival; OS, overall survival; ESCC, esophageal squamous cell carcinoma.

On multivariate analysis, the gender, age, TNM stage, tumor location PD-L1 expression, and the number of lines of previous systemic therapies were not indicative of progression-free survival and overall survival, and previous radiotherapy within 3 months was significantly related to longer PFS and OS (p < 0.05) (**Table 2**). Patients who had received radiotherapy within 3 months prior to immunotherapy had significantly longer progression-free survival with sintilimab than those who did not receive radiotherapy (median progression-free survival 10.0 months [95% CI 8.030–11.970] *vs.* 5.0 months [95% CI 2.755–7.245]) (**Figure 2C**). In contrast, overall survival was significantly longer in patients who had received radiotherapy within 3 months prior to sintilimab than in those who had not (median overall survival 15.3 months [95% CI 13.724–16.876] *vs.* 12.2 months [10.001–14.399]) (**Figure 2D**). Progression events occurred in 44 (49%) of the 89 patients who had received radiotherapy for ESCC within 3 months prior to immunotherapy, compared to 30 (68.2%) of the 44 patients who had not previously received radiotherapy. Therefore, ORR and DCR were higher in the subgroup that had received radiotherapy for ESCC within 3 months prior to immunotherapy (**Table 3**). Our results showed that radiotherapy within 3 months before sintilimab can enhance the efficiency of immunotherapy and benefit the prognosis in unresectable advanced ESCC patients who received multi-line sintilimab.

**Table 2:** Cox regression analyses of factors affecting the PFS and OS of ESCC patients (n = 133).

Variable	PFS		OS	
	p	HR (95% CI)	p	HR (95% CI)
Sex (male/female)	0.823	0.944 (0.568–1.567)	0.342	0.773 (0.454–1.315)
Age (years) (<65/≥65)	0.755	0.927 (0.576–1.492)	0.913	0.972 (0.588–1.609)
T stage	0.906	0.982 (0.721–1.336)	0.196	0.803 (0.575–1.120)
N stage	0.737	0.923 (0.576–1.477)	0.991	0.997 (0.606–1.640)
M stage	0.443	1.216 (0.737–2.006)	0.288	0.758 (0.455–1.264)
Tumor location (upper/middle/lower thoracic)	0.637	1.084 (0.775–1.516)	0.738	1.061 (0.748–1.506)
Number of previous systemic therapies	0.705	0.851 (0.369–1.961)	0.116	3.183 (0.751–13.499)
PD-L1 expression before immunotherapy (negative/positive)	0.826	0.944 (0.562–1.585)	0.075	0.582 (0.321–1.056)
Radiation before immunotherapy within 3 months (yes/no)	**0.006**	0.479 (0.285–0.806)	**0.030**	0.510 (0.278–0.936)

Statistically significant values appear in bold (p < 0.05).

ESCC, esophageal squamous cell carcinoma; PD-L1, programmed cell death protein ligand 1; PFS, progression-free survival; OS, overall survival; HR, hazard ratio.

**Table 3:** Tumor response in advanced ESCC.

Tumor response	All (n = 133)	Received radiotherapy before sintilimab	
		Beyond 3 months or no radiotherapy (n = 44)	Within 3 months (n = 89)
PR	15	2	13
SD	46	12	34
PD	72	30	42
ORR	11.3%	4.5%	14.6%
DCR	45.9%	31.8%	52.8%

ESCC, esophageal squamous cell carcinoma; CR, complete response; PR, partial response; SD, stable disease; PD, disease progression; ORR, objective response rate; DCR, disease control rate.

### 3.3 Efficacy and safety of multi-line sintilimab

Of 133 patients, 15 patients experienced a partial response, and 14 patients had stable disease. The ORR was 11.3% and the DCR was 45.9% (**Table 3**). A total of 72 patients experienced disease progression, and the median PFS with sintilimab treatment was 9.0 months (95% CI 7.701–10.299) (**Figure 2A**). In 66 patients who died, the median overall survival was 14.9 months (95% CI 12.558–17.242) (**Figure 2B**).

All patients were treated with at least two cycles of sintilimab, and no patient was withdrawn from the course of immunotherapy due to toxic effects. AEs of sintilimab in patients were also recorded, such as a white blood cell count decrease (36.8%), anemia (19.5%), rash (9.0%), aminotransferase increase (5.3%), diarrhea (3.0%), and asthenia (0.8%), all common AEs of sintilimab. According to the radiotherapy and immunotherapy interval, 133 patients were divided into two subgroups, and there was no statistical difference in adverse reactions between the two groups (**Table 4**). No immune-related deaths occurred. All adverse reactions were handled appropriately. A total of 66 patients died in this study, and the immediate causes of death were tumor progression. All deaths were determined to be related to immunotherapy.

**Table 4:** Adverse events of subgroup patients (n = 133).

	Received radiotherapy before sintilimab	
	Beyond 3 months or no radiotherapy (n = 44)	Within 3 months (n = 89)
Any event	34 (77.3%)	65 (73.0%)
White blood cell count decreased	13 (29.5%)	36 (40.4%)
Rash	14 (31.8%)	12 (13.5%)
Anemia	5 (11.4%)	7 (7.9%)
Aminotransferase increased	1 (2.3%)	6 (6.7%)
Diarrhea	1 (2.3%)	3 (3.4%)
Asthenia	0 (0%)	1 (1.1%)

## 4 Discussion

In this retrospective study, sintilimab exhibited significant efficacy in unresectable advanced ESCC that had been previously treated, and pre-immunotherapy radiotherapy within 3 months could enhance the efficacy of multi-line sintilimab.

Radiation therapy (RT) has an important place in the treatment of advanced ESCC, providing effective relief of dysphagia and leading to improved long-term survival through enhanced local disease control (13, 14). To avoid overactive immune responses leading to excessive inflammatory responses and autoimmune diseases, the body has evolved immune checkpoint mechanisms to control the intensity and duration of immune responses and minimize the damage of immune responses to healthy tissues, mainly the CTLA4-B7 pathway and PD-1/PD-L1 pathway. However, after tumor cell invasion, tumor cells will use this suppressive pathway to suppress T-cell activation and thus escape the immune system’s siege, a process known as an immune escape (15). The anti-tumor effects of radiotherapy are achieved by directly inducing DNA damage in the target cells. Radiotherapy controls local lesions through direct action and also controls distant metastases by inducing the abscopal effect. A commonly hypothesized theory is that local radiotherapy leads to immunogenic cell death, resulting in an inflammatory microenvironment. This is characterized by the release of tumor antigens and damage-associated pattern molecules (DAMPs) from the dead cells. Radiotherapy also induces the expression of chemokines, leading T cells to the tumor microenvironment (13–15), thus improving efficiency and reducing adverse effects (16–18). However, immune escape often occurs with the recurrence of the tumor, limiting the ability of radiotherapy to produce an anti-tumor immune response (19, 20). Therefore, radiotherapy plays a crucial role in tumor suppression as a bridge between innate and acquired immune responses.

Previous studies have shown the additional efficacy of immunotherapy in combination with RT in solid tumors (19, 20). Postow et al. reported on the first-time tumor regression in a melanoma patient after receiving ipilimumab combined with RT, even outside the radiation area (21). A growing body of clinical evidence suggests that two to three courses of combination therapy (checkpoint inhibitors in combination with RT) have good potential and are well tolerated in patients with a variety of locally advanced or metastatic malignancies, including non-small cell lung cancer (NSCLC), melanoma, and renal cancer. To the best of our knowledge, the present study, which combined short-term radiotherapy and PD-L1 expression status, is the first to evaluate retrospective data on patients with advanced ESCC cancer who received sintilimab and prior radiotherapy and were divided into two subgroups for comparison based on the time interval between radiotherapy and immunotherapy. Most studies have reported that higher PD-L1 expression in EC was related to a poor prognosis; however, high PD-L1 expression patients responded well to anti-PD1/PD-L1 monoclonal antibodies and had significantly higher OS rates (22, 23). These results indicate that PD-L1 expression can be a meaningful biomarker to identify the most optimal population for anti-PD-1/PD-L1 therapy. In our study, although fewer patients with high PD-L1 expression were in the subgroup, these patients who received radiotherapy within 3 months prior to immunotherapy had significantly longer progression-free survival than those who did not receive radiotherapy, suggesting that radiotherapy may have upregulated PD-L1 expression in patients, thereby converting traditional non-responders into responders and contributing to the efficacy of immunotherapy.

After successful clearance of the infecting pathogen or malignant cells, the presence of a population of suppressor immune cells allows the body to restore immune homeostasis to avoid immune damage caused by an excessive immune response. A variety of suppressive immune cells are distributed in the tumor microenvironment, including CD+8T cells, macrophages and myeloid-derived suppressor cells (MDSCs), and other stromal cells. When tumorigenesis occurs, large numbers of immature MDSCs promote tumor invasion and metastasis through accumulation and suppression of T-cell immune function by direct cell-to-cell interactions or secretion of cytokines. Tumor-associated macrophages (TAMs) can as well be initiated by different isoforms and in an environment-dependent manner. An increasing body of research has shown that the quantity of T cells in immune organs increases significantly when patients are exposed to local or systemic radiation (24). A recent study by Dovedi et al. showed that PD-L1 expression was upregulated in tumor cells through interferon-γ production by CD8+ T cells after radiotherapy (25). In addition, radiation therapy can trigger a local inflammatory response while stimulating the induction of PD-L1 expression in the tumor microenvironment and decreasing the sensitivity of the anti-tumor immune response (13). Thus, if the PD-1/PD-L1-induced immune escape mechanism can be overcome, radiotherapy has the potential to have a more sustained effect on tumor cells.

Our study is the first retrospective study to assess the association between a previous history of radiotherapy and the clinical value of sintilimab in patients with advanced ESCC, which may provide evidence for future randomized clinical studies. At the same time, the study has limitations. This was a retrospective study, which may result in selection bias. We observed that pre-immunotherapy radiotherapy resulted in significant differences in PFS and OS, and there was no correlation between PD-L1 expression status from this study. First, relatively few patients were enrolled in our study, and they were all from a single institution. Although our pooled analysis alleviated the sample size issue for each trial, subgroup analysis was still limited by the low sample size, and therefore, a larger sample size is needed to more accurately detect the impact of the combination of radiotherapy and immunotherapy on patient outcomes. There were no uniform criteria for positive PD-L1 expression, which may also have resulted in different outcomes than previous studies. Since this study did not differentiate the efficacy of different treatment modalities, the exact effect of specific treatment regimens could not be determined in this study. We are expanding the sample size to reduce the impact of different treatment options on outcomes.

In conclusion, sintilimab is an option for multi-line treatment in unresectable advanced esophageal squamous cell carcinoma patients, and radiotherapy within 3 months prior to immunotherapy enhances the immune efficacy of sintilimab, resulting in longer PFS and OS.

## Data availability statement

The raw data supporting the conclusions of this article will be made available by the authors, without undue reservation.

## Ethics statement

The study was approved by all participating institutions. The study was conducted in accordance with the Declaration of Helsinki and International Good Clinical Practice Guidelines. All patients provided written informed consent before study participation. 

## Author contributions

SX: conceptualization, data curation, formal analysis, and writing—original draft. XX: data curation, formal analysis, and writing—original draft. HZ: conceptualization, funding acquisition, project administration, and writing—review and editing. All authors contributed to the article and approved the submitted version.
